# Estimated incidence of juvenile-onset recurrent respiratory papillomatosis in Korea

**DOI:** 10.4178/epih.e2021019

**Published:** 2021-03-10

**Authors:** Jin-Kyoung Oh, Hwa Young Choi, Minji Han, Yuh-Seog Jung, Sang Joon Lee, Moran Ki

**Affiliations:** 1 Department of Cancer Control and Population Health, National Cancer Center Graduate School of Cancer Science and Policy, Goyang, Korea; 2 Cancer Risk Appraisal and Prevention Branch, National Cancer Center, Goyang, Korea; 3 Department of Health Sciences, Hanyang University, Seoul, Korea; 4 Department of Otorhinolaryngology, Center for Thyroid Cancer, Research Institute and Hospital, National Cancer Center, Goyang, Korea; 5 Department of Otorhinolaryngology-Head and Neck Surgery, Dankook University College of Medicine, Cheonan, Korea

**Keywords:** Recurrent respiratory papillomatosis, Human papillomavirus, Human papillomavirus vaccine, Korea

## Abstract

**OBJECTIVES:**

Recurrent respiratory papillomatosis (RRP) is caused by human papillomavirus (HPV) types 6 and 11 and is potentially preventable through vaccination. This study estimated the incidence of juvenile-onset RRP before the implementation of the national HPV vaccination program in Korea.

**METHODS:**

We conducted a cohort study using claims data provided by a mandatory insurance program to estimate the incidence of RRP and associated healthcare use. Patients with juvenile RRP were defined as those aged ≤12 years with ≥2 admissions or ≥2 outpatient visits during which they received the International Classification of Diseases, 10th revision code for benign neoplasms of the larynx (D14.1).

**RESULTS:**

During 2002-2014, 123 children (74 boys and 49 girls) were diagnosed with RRP. The patients had a mean of 6.5 person-years of follow-up. The incidence was estimated at 0.30/100,000 person-years. The median age at diagnosis was 4.0 years (mean, 4.3). Thirty-six (29.3%) patients underwent surgery, including 23 patients (18.7%) who underwent 2 or more surgical procedures. Severe disease, measured by more frequent surgical procedures and shorter time intervals between consecutive operations, was associated with a younger age at diagnosis.

**CONCLUSIONS:**

The estimated incidence of juvenile-onset RRP in Korea was similar to that reported in other countries. The RRP burden should continue to be monitored using National Health Insurance Service claims data.

## INTRODUCTION

Recurrent respiratory papillomatosis (RRP) is a benign but potentially troublesome disease caused by human papillomavirus (HPV) that is characterized by the appearance of papillomatous lesions in the respiratory tract [1]. HPV types 6 and 11 are responsible for more than 90% of RRP cases [2,3]. RRP is usually restricted to the larynx, but it infrequently becomes aggressive, and spreads to the nasopharynx, tracheobronchial tree, and more rarely, the pulmonary parenchyma [4-6]. The natural course of the disease is diverse and difficult to predict, ranging from spontaneous remission to aggressive progression, which may result in obstruction or spread to the lungs and require multiple surgical procedures to maintain an open airway [7].

Although rare, RRP occurs more commonly in children than in adults. The incidence of RRP has been reported to range from 0.17 to 1.34 per 100,000 in children [8-13] and 0.18 to 0.54 per 100,000 in adults [12,14]. A maternal history of genital warts during pregnancy and delivery is strongly associated with the development of juvenile-onset RRP [15].

The course of the disease can vary from mild to severe. Although no conclusive cure for RRP exists, surgical excision of the papillomas is the primary form of treatment used to ensure functioning of the airway and maintain quality of phonation. Cidofovir, an analog of cytosine, is currently the most commonly used antiviral agent in adjuvant treatment of RRP [16]. Nevertheless, some children require multiple surgical procedures throughout childhood, which are associated with progressively greater physical, emotional, and financial burdens on the individual, family, and society [17,18].

RRP is preventable through appropriate vaccination (i.e., quadrivalent or non-avalent HPV vaccines, which have preventive effects against HPV 6 and 11). HPV vaccines have been used in the private sector in Korea since 2007 to prevent cervical cancer and other HPV-related diseases. In 2016, bivalent and quadrivalent HPV vaccines targeting 12-year-old girls were added to the fully funded National Immunization Program [19]. However, little is known about the epidemiological characteristics of juvenile RRP in Korea. This study estimated the incidence and analyzed the demographic characteristics of Korean children diagnosed with juvenile-onset RRP prior to the introduction of the HPV vaccination program in Korea.

## MATERIALS AND METHODS

### Data source

This study used health insurance claims data provided by the National Health Insurance Service (NHIS). The NHIS is a compulsory, single-payer insurance scheme that provides benefits for the prevention, diagnosis, and treatment of disease and injury. All Korean citizens are required to either be enrolled in the NHIS (97% of the entire population) or registered for Medical Aid (3%) [20]. The NHIS database includes information on Medical Aid recipients; therefore, it covers the entire Korean population. Currently, the NHIS maintains and stores national records for healthcare service use and prescriptions. The NHIS claims database contains details on diagnoses, treatments and medical services, prescriptions, number of outpatient visits, length of hospitalization, cost of care, the medical institution, as well as the income level and place of residence of all insured individuals [21].

### Study population

The incident RRP patients were defined as those aged 12 years and younger with ≥2 admissions or ≥2 outpatient visits during which they were assigned the International Classification of Diseases, 10th revision (ICD-10) code corresponding to benign neoplasms of the larynx (D14.1). In a study conducted in Australia, the positive predictive value of code D14.1 for RRP was 98.1% [11]. The RRP cohort consisted of patients born in or after 2002 who were first diagnosed with RRP between 2002 and 2014. The RRP cohort was followed-up until December 31, 2015. Their use of medical services, such as the number of hospital visits and surgical treatments, was the outcome of interest. The incidence rate was calculated as the total number of cases divided by total person-year (i.e., the mid-year population of children born between 2002 and 2014 [22] multiplied by the follow-up years).

### Ethics statement

The study protocol was approved by the Institutional Review Board (IRB) of the National Cancer Center of Korea (NCC2017-0034). This study used secondary data that contained no personal information, and the requirement for informed consent was waived by the IRB.

## RESULTS

**Figure 1.**
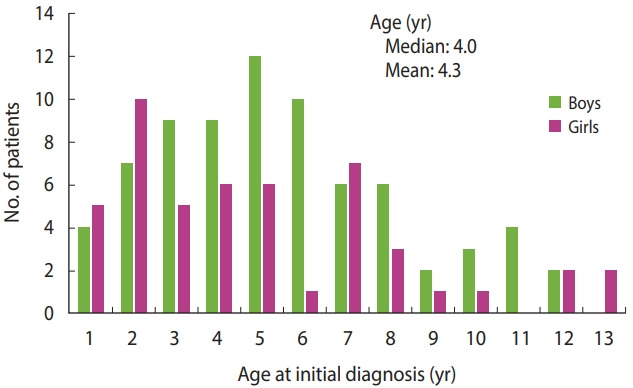
Patients with juvenile-onset recurrent respiratory papillomatosis among the birth cohort born between 2002 and 2014 stratified by age at initial diagnosis in Korea (n=123).

Table 1 and Figure 1 show the demographic characteristics of patients with juvenile RRP diagnosed between 2002 and 2014. A total of 123 children (74 boys and 49 girls) aged 12 years or younger were diagnosed with RRP. The estimated incidence rate was 0.30 per 100,000 person-years. The median age at diagnosis was 4.0 years (mean, 4.3); most patients (78.8%) were under the age of 6 years at the time of the initial diagnosis.

Table 2 shows the age distribution of patients with incident RRP (n=123) and patients who had undergone surgery 2 or more times (n=23). The mean age of patients who underwent ≥2 surgical procedures was 2.6 years.

**Figure 2.**
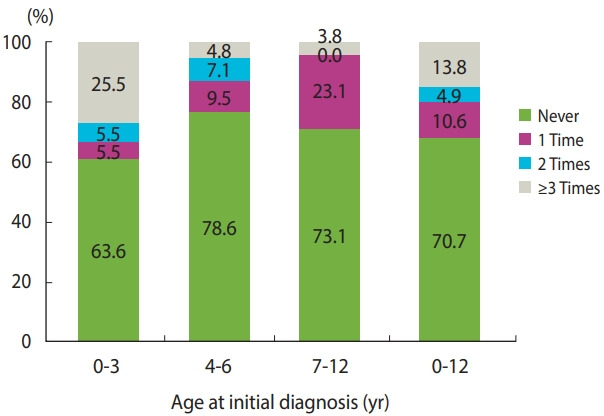
Patients with juvenile-onset recurrent respiratory papillomatosis stratified by frequency of surgical intervention and age at initial diagnosis in Korea (n=123).

Figure 2 shows the distribution of patients with RRP by frequency of surgical intervention. Thirty-six patients (29.3%) received a surgical intervention for RRP, including 23 patients (18.7%) who underwent surgery 2 or more times. The patients diagnosed at a younger age were more likely to have received repeated surgical interventions, with repeated surgery rates of 30.9%, 11.9%, and 3.8% in the age groups of ≤3 years, 4-6 years, and 7-12 years, respectively. The remaining 70.7% of patients never received a surgical intervention. The incidence density of patients who received surgical interventions was 5.1 per 100 person-years.

Table 3 shows the time interval between surgical procedures or outpatient visits. The time interval between subsequent surgical procedures was shorter in the age group ≤3 years at initial diagnosis than in the older age group. In contrast, for patients treated without surgery, the time interval between the first visit for diagnosis and the following visits for treatment or follow-up was shorter in the older age group. Among 36 patients who received surgical interventions, 21 (58.3%) underwent repeated surgery within 1 year (data not shown).

As shown in Table 4, the most commonly applied surgical interventions were resection of the laryngeal benign tumor under suspension laryngoscopy, followed by removal of the vocal nodule or polyp, endoscopic dilation of tracheal or bronchial stenosis with balloon, operation of laryngeal stenosis (laser operation), operation of vocal cord paralysis (unilateral, foreign material injection, Teflon, silicone, or other), removal of epiglottic cyst (n=4), diffuse vocal polyposis incision and suction, resection of the laryngeal benign tumor under flexible endoscopy, and endoscopic excision of tracheal or bronchial tumor (rigid bronchoscopic).

## DISCUSSION

In this study, we found that juvenile-onset RRP is rare in Korea, with an annual incidence rate of 0.30 per 100,000 person-year among those aged ≤12 years. This incidence rate is comparable with the RRP incidence reported in other countries for a similar age group. For example, in a population-based study conducted in the United States, the estimated incidence rate for juvenile RRP was 0.3 to 1.1 per 100,000 children aged <18 years in 1996 and the prevalence rate was 1.7 to 2.6 per 100,000 children [8]. In a 2006 study in the United States, the incidence and prevalence were 0.5 to 1.0 and 1.5 to 2.9 per 100,000 children (<18 years), respectively [10]. In a Canadian national database of children with juvenile RRP, recorded during 1994-2007, the incidence rate was 0.24 per 100,000 children aged ≤14 years and the prevalence was 1.1 per 100,000 children in the same age group [9]. In Australia, during 2000-2013, the estimated national prevalence rate was 0.81 per 100,000 children aged <15 years, peaking within the age range of 5-9 years (1.1 per 100,000) [11]. A study conducted in South Africa estimated the incidence and prevalence rates of juvenile RRP in South Africa at 1.3 and 3.9 per 100,000 children aged 14 years and younger, respectively [13]. In our study, the median age at diagnosis was 3 years, which is comparable to that in the United States (3.1 years) [17] and Australia (3.0 years) [11].

Although no conclusive cure for RRP exists, surgical excision of the papillomas is the primary form of treatment [16]. Among the children in our study, 18.7% had experienced repeated surgical interventions during an average of 6 years of follow-up; patients diagnosed at a younger age were more likely to receive repeated surgical interventions. Younger age at onset of juvenile RRP is an important predictor of severity. According to a systematic review, younger age at onset has consistently been related to disease severity and is one of the most important risk factors for severity [15]. However, in a United States-based study using the national registry data of juvenile-onset RRP, severity (as measured by frequency of surgical procedures) was more strongly associated with age at the time of the first surgery than age at the time of diagnosis [17]. Buchinsky et al. [23] reported that the probability of aggressive disease is high for children under 5 years of age and then drops rapidly. HPV type was not significantly associated with aggressiveness.

A systematic review grouped the major risk factors for juvenile-onset RRP into 3 categories: history of maternal genital HPV infection and birth, viral genotype, and host factors [15]. HPV infection is a major risk factor for juvenile-onset RRP. A history of maternal genital warts during pregnancy and delivery is strongly associated with the development of juvenile-onset RRP [15]. One large study concluded that 37% of juvenile-onset RRP cases were associated with maternal genital warts during pregnancy [24]. HPV types 6 and 11 are the cause of more than 90% of RRP cases [7,25], with HPV 11 associated with a higher severity. In previous studies, young age at diagnosis and infection with HPV 11 were the most important risk factors for severity in juvenile-onset RRP [15]. In our study, we found that surgery was more frequent among patients who were younger at diagnosis; in addition, the time between consecutive operations was shorter in the younger age group.

HPV infection is preventable through vaccination. In 2007, the quadrivalent HPV vaccine (HPV 6, 11, 16, and 18) was approved in Korea for girls and women aged between 9 years and 26 years and men aged between 9 years and 15 years. Since 2011, it has also been indicated for men and women aged between 9 years and 26 years. In 2008, the bivalent HPV vaccine (HPV 16 and 18), which does not protect against the HPV types associated with RRP, was approved for use in girls and women aged between 9 years and 25 years. In 2014, a 2-dose vaccination schedule was approved for use in girls and boys aged between 9 years and 13 years (quadrivalent) and for girls aged between 9 years and 14 years (bivalent). The non-valent HPV vaccine (HPV 6, 11, 16, 18, 31, 33, 45, 52, and 58) was licensed in 2016 [26]. In June 2016, vaccination against bivalent and quadrivalent HPV was included in the National Immunization Program as 2-dose schedule vaccines (0 and 6 months) for girls aged 12 years. The choice of vaccine is based on the clinician’s recommendation and patient and/or parent preferences [19]. The HPV vaccination rate for 12-year-old girls in 2018 was 87.2% [27]. The quadrivalent HPV vaccine, which protects against HPV 6 and 11, is widely used in Korea, with an estimated 80% of the vaccinated population having received it (internal data of the Korea Centers for Disease Control and Prevention). The effects of vaccination on RRP are likely to become apparent in the future, once vaccinated girls grow to women of child-bearing age. Following an extensive quadrivalent HPV vaccination program in Australia, the average annual incidence rate of juvenile-onset RRP declined from 0.16 per 100,000 in 2012 to 0.02 per 100,000 in 2016 [28].

This study has potential limitations. First, the estimated incidence was based only on patients who visited medical facilities because we used health insurance claims data. However, since the NHIS database contains information on the entire Korean population, cases of children with RRP who did not receive treatment are likely to be extremely rare.

Second, we used ICD-10 code D14.1 to identify RRP cases, which may have provided an inaccurate estimate, as several other codes can be used to designate RRP, depending on context. For example, according to a United Kingdom-based study, the ICD-10 codes corresponding to an RRP episode can include D10 (benign neoplasm of mouth and pharynx), D14.1 (benign neoplasm of larynx), D14.2 (benign neoplasm of trachea), D14.3 (benign neoplasm of bronchus and lung), D14.4 (benign neoplasm of unspecified respiratory system), and B97.7 (papillomavirus as a cause of diseases classified in other chapters) [29]. It has been suggested that J38 (diseases of vocal cords and larynx, not elsewhere classified) and J383 (other diseases of vocal cords) should be included in RRP coding in Korea. However, at the time of the study was performed, codes other than D14.1 were being used to denote other conditions, such as polyps of the airway, as well as RRP. For example, in this study, the number of patients aged 12 years or younger with J38 and J383 exceeded 12,000 and 134,000, respectively. When we combined these disease codes (J38/J383) with the treatment code for the most commonly used surgical intervention for RRP (O1221: resection of laryngeal benign tumor under suspension laryngoscopy), we obtained implausibly many (J38 & O1221=722) or few (J383 & O1221=0) cases. Nonetheless, D14.1 can also be used for other non-papilloma conditions (e.g., polyps in the airway), which can lead to overestimation of RRP. Additionally, we excluded patients diagnosed with D14.1 just once to ensure that cases met the definition of “recurrent.” The number of RRP cases can be increased by extending the observational period prospectively. For example, children born in 2014 and diagnosed with D14.1 once were excluded in this study. The follow-up time is very short for them (i.e., 1 year), and they will become RRP cases if D14.1 recurs in the future.

In this study, 70% of patients did not receive surgical interventions, which suggests that the incidence of RRP may have been overestimated. Some of them might not have been true RRP cases, while others might have been treated with the wait-and-see approach recommended for RRP with mild to moderate symptoms, or received treatment not covered by the NHIS. In Korea, the coding used to denote RRP is likely diverse, as RRP is a very rare disease and there are no RRP-specific diagnosis codes or standardized guidelines for diagnosis and treatment. Additionally, regardless of the underlying diagnosis with any ICD-10 codes, we observed 200 patients who had received 1 of the 4 surgical interventions that are most commonly applied for RRP (i.e., O1221, resection of laryngeal benign tumor under suspension laryngoscopy; O1231, removal of vocal nodule or polyp; OA273, operation of laryngeal stenosis – laser operation; or O1233, diffuse vocal polyposis incision and suction) (data not shown). Among them, the most common diagnosis was benign neoplasm of the larynx (D14.1, 354 cases), followed by stenosis of the larynx (J38.6, 183 cases), polyp of vocal cords and larynx (J38.1, 81 cases), nodules of the vocal cords (J38.2, 34 cases), and congenital subglottic stenosis (Q31.1, 32 cases) (The total number of cases is larger than the number of patients because some patients had more than 1 type of lesion). Despite the risk of over-estimation or under-estimation associated with using a single, non-specific disease code, the findings from this study suggest that D14.1 is a sensitive and specific ICD-10 code for juvenile-onset RRP in Korea. In an Australia-based study, the positive predictive value of code D14.1 was 98.1% [11]. Applying the stringent criterion of a history of 2 or more surgical procedures explicitly due to RRP, the total incidence reported is 23. However, when applying the estimation method described previously, the estimated incidence of RRP is 123. For purposes of clarity, both total incidence values are presented in Table 2.

Third, data on the use of medical adjuvant treatment were not available in this study. Approximately 20% of patients with RRP require adjunctive medical treatment in addition to surgery to control the disease [16,25]. The current criteria for adjuvant therapy include more than 4 surgical procedures annually, rapid recurrence of papillomas with airway compromise, and distal multisite spread of the disease. The medications used in treatment include interferon, antiviral agents (acyclovir, ribavirin, cidofovir), retinoids, and inhibitors of the oxygenase-2 cycle [16,25]. Cidofovir, an analog of cytosine, is currently the most commonly used antiviral agent in medical adjuvant treatment for RRP [1,16]. However, most medications used for RRP, including cidofovir, are not covered by the NHIS, and claims data are not available for these medications.

Lastly, clinical information including pathology, which is useful for confirming the diagnosis, is unavailable from the claims data.

Despite these limitations, this study provides valuable information on the epidemiological features and treatment modality of RRP in Korea. Future studies on the epidemiology of juvenile-onset RRP after the implementation of the national HPV vaccination program are needed. Special coding for RRP claims and/or RRP registration can be suggested for further studies.

In conclusion, the estimated incidence of juvenile the estimated incidence of juvenile-onset RRP in Korea was similar to that reported in other countries. Disease severity, as measured by more frequent surgical procedures and shorter time intervals between consecutive operations, is linked to a younger age at diagnosis. The RRP burden following the implementation of a national HPV vaccination program can be monitored using the NHIS claims data.

## Figures and Tables

**Table 1.** Structure of the recurrent respiratory papillomatosis (RRP) patient cohort

Birth year	Children ever born (thousand)	RRP patient cohort													Age at initial diagnosis (no. of patients)
		No. of patients initially diagnosed per year													
		2002	2003	2004	2005	2006	2007	2008	2009	2010	2011	2012	2013	2014	
2002	470.3	2	0	0	1	0	0	2	2	0	0	2	0	0	-
2003	469.3	-	3	0	2	1	2	3	1	0	1	0	3	1	12 (0)
2004	451.2	-	-	0	0	2	2	1	2	3	2	0	2	0	11 (1)
2005	413.9	-	-	-	2	1	2	3	2	1	1	2	1	0	10 (5)
2006	424.7	-	-	-	-	4	0	2	1	3	1	2	2	3	9 (2)
2007	470.3	-	-	-	-	-	2	0	2	1	2	2	1	1	8 (5)
2008	444.4	-	-	-	-	-	-	7	1	1	3	0	1	0	7 (9)
2009	424.5	-	-	-	-	-	-	-	3	3	2	0	0	1	6 (10)
2010	448.5	-	-	-	-	-	-	-	-	1	2	0	0	0	5 (11)
2011	451.6	-	-	-	-	-	-	-	-	-	1	0	2	1	4 (10)
2012	464.4	-	-	-	-	-	-	-	-	-	-	1	2	1	3 (13)
2013	421.5	-	-	-	-	-	-	-	-	-	-	-	3	1	2 (16)
2014	419.8	-	-	-	-	-	-	-	-	-	-	-	-	2	1 (10)
														-	0 (31)
Total		2	3	0	5	8	8	18	14	13	15	9	17	11	123

**Table 2.** Patients with juvenile-onset recurrent respiratory papillomatosis among the birth cohort born between 2002 and 2014 stratified by age at initial diagnosis in Korea

Age at initial diagnosis (yr)	Incident RRP patients^1^			Patients who underwent 2 or more surgical procedures		
	Boys	Girls	Total	Boys	Girls	Total
Mean±SD [range]	4.5±2.8 [0-11]	3.9±3.3 [0-12]	4.3±3.0 [0-12]	2.1±1.6 [0-5]	3.2±3.1 [1-11]	2.6±2.4 [0-11]
Median (Q1, Q3)	4 (2, 6)	3 (1, 6)	4 (2, 6)	2 (1, 3)	2 (1, 4)	2 (1, 4)
0	4 (5.4)	5 (10.2)	9 (7.3)	1 (8.3)	0 (0.0)	1 (4.3)
1	7 (9.5)	10 (20.4)	17 (13.8)	4 (33.3)	5 (45.5)	9 (39.1)
2	9 (12.2)	5 (10.2)	14 (11.4)	4 (33.3)	1 (9.1)	5 (21.7)
3	9 (12.2)	6 (12.2)	15 (12.2)	1 (8.3)	1 (9.1)	2 (8.7)
4	12 (16.2)	6 (12.2)	18 (14.6)	0 (0.0)	2 (18.2)	2 (8.7)
5	10 (13.5)	1 (2.0)	11 (8.9)	2 (16.7)	0 (0.0)	2 (8.7)
6	6 (8.1)	7 (14.3)	13 (10.6)	0 (0.0)	1 (9.1)	1 (4.3)
7	6 (8.1)	3 (6.1)	9 (7.3)	0 (0.0)	0 (0.0)	0 (0.0)
8	2 (2.7)	1 (2.0)	3 (2.4)	0 (0.0)	0 (0.0)	0 (0.0)
9	3 (4.1)	1 (2.0)	4 (3.3)	0 (0.0)	0 (0.0)	0 (0.0)
10	4 (5.4)	0 (0.0)	4 (3.3)	0 (0.0)	0 (0.0)	0 (0.0)
11	2 (2.7)	2 (4.1)	4 (3.3)	0 (0.0)	1 (9.1)	1 (4.3)
12	0 (0.0)	2 (4.1)	2 (1.6)	0 (0.0)	0 (0.0)	0 (0.0)
Total	74 (100)	49 (100)	123 (100)	12 (100)	11 (100)	23 (100)

Values are presented as number (%). SD, standard deviation.

1 Defined as those aged 12 years and younger with ≥2 admissions or ≥2 outpatient visits during which they were assigned the International Classification of Dis eases, 10th revision code corresponding to benign neoplasms of the larynx (D14.1).

**Table 3.** Average time interval (days) between hospital visits among patients with recurrent respiratory papillomatosis by age group

Surgical procedure	Age at initial diagnosis, yr		
	0-3 (n=55)	4-6 (n=42)	7-12 (n=26)
Patients with surgical intervention, n	20	9	7
Diagnosis to first surgery	46.0 [0-560]	67.9 [0-245]	103.3 [0-694]
First to second surgery	95.5 [7-319]	160.4 [31-274]	14.0 [14-14]
Second to third surgery	129.1 [33-336]	326.5 [322-331]	280.0 [280-280]
Patients without surgical intervention, n	35	33	19
Diagnosis to second visit	93.5 [0-764]	86.4 [6-510]	35.9 [3-191]
Second to third visit	203.1 [10-1,071]	96.3 [11-350]	45.5 [0-189]
Third to fourth visit	130.7 [1-189]	56.0 [56-56]	76.8 [6-210]

Values are presented as day [range].

**Table 4.** List of commonly applied surgical interventions (procedure codes) for juvenile-onset recurrent respiratory papillomatosis during 2002-2014 in Korea

Surgical intervention	Frequency (no. of patients)^1^
Resection of laryngeal benign tumor under suspension laryngoscopy	86 (23)
Removal of vocal nodule or polyp	73 (12)
Endoscopic dilation of tracheal or bronchial stenosis with balloon	15 (2)
Operation of laryngeal stenosis – laser operation	6 (4)
Operation of vocal cord paralysis – unilateral, foreign material injection	4 (4)
Removal of epiglottic cyst	3 (3)
Diffuse vocal polyposis incision and suction	3 (2)
Resection of laryngeal benign tumor under flexible endoscopy	2 (1)
Endoscopic excision of tracheal or bronchial tumor – rigid bronchoscopic	3 (3)

1 Surgical interventions with a frequency ≤2 are not listed.

## References

[1] Carifi M, Napolitano D, Morandi M, Dall’Olio D (2015). Recurrent respiratory papillomatosis: current and future perspectives. Ther Clin Risk Manag.

[2] Gillison ML, Alemany L, Snijders PJ, Chaturvedi A, Steinberg BM, Schwartz S (2012). Human papillomavirus and diseases of the upper airway: head and neck cancer and respiratory papillomatosis. Vaccine.

[3] Omland T, Lie KA, Akre H, Sandlie LE, Jebsen P, Sandvik L (2014). Recurrent respiratory papillomatosis: HPV genotypes and risk of high-grade laryngeal neoplasia. PLoS One.

[4] Ağgünlü L, Erbaş G (2009). Recurrent respiratory papillomatosis with lung involvement. Diagn Interv Radiol.

[5] Chang CH, Wang HC, Wu MT, Lu JY (2006). Virtual bronchoscopy for diagnosis of recurrent respiratory papillomatosis. J Formos Med Assoc.

[6] Marchiori E, Araujo Neto Cd, Meirelles GS, Irion KL, Zanetti G, Missrie I (2008). Laryngotracheobronchial papillomatosis: findings on computed tomography scans of the chest. J Bras Pneumol.

[7] Fusconi M, Grasso M, Greco A, Gallo A, Campo F, Remacle M (2014). Recurrent respiratory papillomatosis by HPV: review of the literature and update on the use of cidofovir. Acta Otorhinolaryngol Ital.

[8] Armstrong LR, Preston EJ, Reichert M, Phillips DL, Nisenbaum R, Todd NW (2000). Incidence and prevalence of recurrent respiratory papillomatosis among children in Atlanta and Seattle. Clin Infect Dis.

[9] Campisi P, Hawkes M, Simpson K, Canadian Juvenile Onset Recurrent Respiratory Papillomatosis Working Group (2010). The epidemiology of juvenile onset recurrent respiratory papillomatosis derived from a population level national database. Laryngoscope.

[10] Marsico M, Mehta V, Chastek B, Liaw KL, Derkay C (2014). Estimating the incidence and prevalence of juvenile-onset recurrent respiratory papillomatosis in publicly and privately insured claims databases in the United States. Sex Transm Dis.

[11] Novakovic D, Cheng AT, Baguley K, Walker P, Harrison H, Soma M (2016). Juvenile recurrent respiratory papillomatosis: 10-year audit and Australian prevalence estimates. Laryngoscope.

[12] Omland T, Akre H, Vårdal M, Brøndbo K (2012). Epidemiological aspects of recurrent respiratory papillomatosis: a population-based study. Laryngoscope.

[13] Seedat RY (2014). The incidence and prevalence of juvenile-onset recurrent respiratory papillomatosis in the Free State province of South Africa and Lesotho. Int J Pediatr Otorhinolaryngol.

[14] Seedat RY, Schall R (2018). Age of diagnosis, incidence and prevalence of recurrent respiratory papillomatosis- a South African perspective. Clin Otolaryngol.

[15] Niyibizi J, Rodier C, Wassef M, Trottier H (2014). Risk factors for the development and severity of juvenile-onset recurrent respiratory papillomatosis: a systematic review. Int J Pediatr Otorhinolaryngol.

[16] Fortes HR, von Ranke FM, Escuissato DL, Araujo Neto CA, Zanetti G, Hochhegger B (2017). Recurrent respiratory papillomatosis: a state-of-the-art review. Respir Med.

[17] Reeves WC, Ruparelia SS, Swanson KI, Derkay CS, Marcus A, Unger ER (2003). National registry for juvenile-onset recurrent respiratory papillomatosis. Arch Otolaryngol Head Neck Surg.

[18] Venkatesan NN, Pine HS, Underbrink MP (2012). Recurrent respiratory papillomatosis. Otolaryngol Clin North Am.

[19] Korea Centers for Disease Control and Prevention Guidelines for the National Immunization Program 2017 [cited 2019 Aug 27]. https://nip.kdca.go.kr/irgd/index.html.

[20] National Health Insurance Service, Health Insurance Review & Assessment Service (2016). National health insurance statistical yearbook.

[21] Seong SC, Kim YY, Khang YH, Heon Park J, Kang HJ, Lee H (2017). Data resource profile: the National Health Information Database of the National Health Insurance Service in South Korea. Int J Epidemiol.

[22] Korean Statistical Information Service Population statistics based on resident registration [cited 2018 Jul 16]. http://kosis.kr/eng/statisticsList/statisticsList_01List.jsp?vwcd=MT_ETITLE&parentId=A.

[23] Buchinsky FJ, Valentino WL, Ruszkay N, Powell E, Derkay CS, Seedat RY (2019). Age at diagnosis, but not HPV type, is strongly associated with clinical course in recurrent respiratory papillomatosis. PLoS One.

[24] Silverberg MJ, Thorsen P, Lindeberg H, Grant LA, Shah KV (2003). Condyloma in pregnancy is strongly predictive of juvenile-onset recurrent respiratory papillomatosis. Obstet Gynecol.

[25] Katsenos S, Becker HD (2011). Recurrent respiratory papillomatosis: a rare chronic disease, difficult to treat, with potential to lung cancer transformation: apropos of two cases and a brief literature review. Case Rep Oncol.

[26] Ministry of Food and Drug Safety Information on pharmaceutical products -- human papillomavirus vaccine [cited 2018 Oct 15]. https://ezdrug.mfds.go.kr.

[27] Korea Centers for Disease Control and Prevention For girls born in 2006-2007, don’t forget to get ‘human papillomavirus (HPV) vaccine’ during summer vacation [cited 2019 Aug 27]. https://nip.kdca.go.kr/irgd/index.html.

[28] Novakovic D, Cheng AT, Zurynski Y, Booy R, Walker PJ, Berkowitz R (2018). A prospective study of the incidence of juvenile-onset recurrent respiratory papillomatosis after implementation of a national HPV vaccination program. J Infect Dis.

[29] Donne AJ, Keltie K, Cole H, Sims AJ, Patrick H, Powell S (2017). Prevalence and management of recurrent respiratory papillomatosis (RRP) in the UK: cross-sectional study. Clin Otolaryngol.

